# The impact of surgeon and patient treatment preferences in an orthopaedic trauma surgery trial

**DOI:** 10.1186/s13063-019-3631-x

**Published:** 2019-09-18

**Authors:** Ada Keding, Helen Handoll, Stephen Brealey, Laura Jefferson, Catherine Hewitt, Belen Corbacho, David Torgerson, Amar Rangan

**Affiliations:** 1 York Trials Unit, Department of Health Sciences, University of York, Heslington, YO10 5DD UK; 2 School of Health and Social Care, Teesside University, Middlesbrough, Tees Valley TS1 3BA UK; 3 Department of Health Sciences, University of York, Heslington, YO10 5DD UK; 4 Department of Trauma and Orthopaedics, James Cook University Hospital, South Tees Hospitals NHS Trust, Marton Road, Middlesbrough, TS4 3BW UK

**Keywords:** Randomised controlled trial, Equipoise, Surgeons, Patient preference, Bias, Validity, Orthopaedic, Non-operative, Surgery, Proximal humeral fractures

## Abstract

**Background:**

Surgeon and patient treatment preferences are important threats to the internal and external validity of surgical trials such as PROFHER, which compared surgical versus non-surgical treatment for displaced fractures of the proximal humerus in adults. We explored the treatment preferences expressed by surgeons and patients in the trial and how these impacted on patient selection, trial conduct and patient outcome.

**Methods:**

A series of exploratory secondary analyses of the PROFHER trial data were undertaken. We reviewed the extent of surgeon and patient treatment preferences (surgery or not surgery) at screening (*n* = 1250) as well as prior preference (including no preference) of randomised patients (*n* = 250), and assessed their impact on recruitment and adherence to follow-up and rehabilitation. Changes in treatment after 2 years’ follow-up were explored. Patient preference and characteristics associated with trial inclusion or treatment preference (*t* test, chi-squared test, Wilcoxon rank-sum test) were included as treatment interaction terms in the primary trial analysis of shoulder functioning (Oxford Shoulder Score, OSS).

**Results:**

Surgeons excluded 17% of otherwise eligible patients based on lack of equipoise; these patients had less complex fractures (*p* < 0.001) and tended to be older (*p* = 0.062). Surgeons were more likely to recommend surgery for patients under 65 years of age (*p* = 0.059) and who had injured their right shoulder (*p* = 0.052). Over half of eligible patients (56%) did not consent to take part in the trial; these patients tended to be older (*p* = 0.022), with a preference for not surgery (74%; which was associated with older age, *p* = 0.039). There were no differential treatment effects (*p* value of interaction) for shoulder functioning (OSS) based on subgroups of patient preference (*p* = 0.751), age group (*p* = 0.264), fracture type (*p* = 0.954) and shoulder dominance (*p* = 0.850). Patients who were randomised to their preferred treatment had better follow-up rates (94 vs 84% at 2 years) and treatment adherence (90 vs 83% reported completing home exercises). Patients who were not randomised to their preferred treatment were more likely to change their treatment preference at 24 months (60 vs 26%).

**Conclusions:**

The robustness of the PROFHER trial findings was confirmed against possible bias introduced by surgeon and patient preferences. The importance of collecting preference data is highlighted.

**Trial registration:**

ISRCTN50850043. Registered on 25 March 2008.

## Background

Surgeon and patient treatment preferences are two major challenges to the success and integrity of randomised controlled trials (RCTs) of surgery, especially of sharply contrasting interventions where treatment preferences are typically stronger and blinding impractical [1, 2]. Both can seriously affect: trial recruitment, sometimes to the abandonment of the trial [3]; acceptance of allocated treatment, with high rates of crossover putting the trial in jeopardy [4]; adherence to allocated treatments and follow-up; and outcome assessment [5, 6]. Strong treatment preferences can seriously impact on both the internal and external validity of the trial; the second often reflecting important clinical disparity between the intended and the actual trial population [7]. Even where the trial has been completed successfully, preferences among other factors can still present a strong barrier to the dissemination and implementation of trial results [8].

Against this background, detailed preference data were collected from surgeons and patients at different stages of the PROximal Fracture of the Humerus Evaluation by Randomisation (PROFHER) trial. PROFHER was a pragmatic, multi-centre RCT, funded by the National Institute for Health Research (NIHR) (UK), that compared surgical with non-surgical treatment in adults with displaced fractures of the proximal humerus involving the surgical neck [9, 10]. The protocol target of 250 adults was recruited between September 2008 and April 2011 from 33 acute care NHS hospitals in the UK. Over 2 years’ follow-up, there was no significant difference between surgical compared with non-surgical treatment in patient-reported shoulder functioning (Oxford Shoulder Score, OSS). PROFHER concluded that the trial’s results do not support the trend of increased surgery for patients with these fractures [10, 11]. The direct applicability of PROFHER’s findings to current UK practice for the management of displaced proximal humeral fractures in NHS hospitals was highlighted in recent National Institute for Health and Care Excellence (NICE) guidelines [12].

Surgeons who were not in equipoise with respect to the study population and trial treatments, as well as patient preferences for either surgery or not surgery, may have impacted on the trial in a number of ways. Preferences may have affected recruitment rate (the trial required a 13-month extension to recruit its target) and influenced the selection of patients into the trial, potentially affecting the generalisability of the trial results (notwithstanding the accepted overall representativeness of the population evidenced by inclusion in NICE guidance [12]). In addition, prior expectations have the potential to influence outcome assessments at follow-up. This article therefore explores the evidence for bias resulting from surgeon and patient treatment preferences and their impact on the validity of the results in the PROFHER trial. We discuss our findings in a more general context of PROFHER and other multi-centre RCTs comparing surgical and non-surgical treatment in orthopaedics.

Using data collected from patients and surgeons throughout the trial, we set out the following research objectives:

Surgeon preferences

- To explore to what extent surgeon treatment preferences affected the selection of eligible patients into the trial and assess the differences in patient characteristics between included and excluded patient populations
- To explore what patient characteristics were associated with surgeon preference for surgery and not surgery for inappropriately excluded patients

Patient preferences

c) To explore to what extent patient treatment preferences affected patients’ willingness to consent to taking part in the trial and assess the differences in patient characteristics between included and excluded patient populations
d) To explore what patient characteristics were associated with patient preference for surgery and for not surgery for non-consenting patients
e) To explore whether patient preferences changed over the course of the trial

Impact

f) To explore whether treatment preferences at baseline affected adherence to the trial treatments and follow-up rates
g) To explore whether patient preferences were associated with treatment differences for the trial’s primary outcome
h) To explore whether differences in patient characteristics associated with preference based patient exclusion as identified under objectives (a) to (d) affected treatment differences for the trial’s primary outcome

## Methods

The methods described here pertain to the analysis of surgeon and patient treatment preferences in the PROFHER trial. A full account of the design and methods of PROFHER is given in the primary trial publication [10].

### Design

This study used data collected as part of the PROFHER trial to explore the relationships between treatment preferences, patient characteristics and outcome assessments.

### Study population

Adult patients with a displaced fracture of the humerus involving the surgical neck who were screened for eligibility for inclusion in the PROFHER trial by an orthopaedic surgeon in fracture clinics and orthopaedic wards of 33 hospitals in the UK.

### Selection criteria

Well-known limitations for patient selection based on the Neer classification [13, 14] were accommodated in PROFHER by using pragmatic inclusion criteria see (Table 1), with emphasis on the individual surgeon’s equipoise in terms of whether or not the surgical-neck fracture should be treated surgically. To counter learning-curve problems and to maximise the number of surgeons in equipoise, surgeons were expected to use surgical interventions and procedures with which they were familiar. Availability of good quality and comparable rehabilitation to all trial participants was ensured [10, 15].

**Table 1:** PROFHER inclusion and exclusion criteria

Inclusion criteria	
Adults (aged 16 years or above) presenting within 3 weeks of their injury with a radiologically confirmed displaced fracture of the humerus involving the surgical neck	
This should include all 2-part surgical-neck fractures; 3-part (including surgical neck) and 4-part fractures of proximal humerus (Neer classification). It may also include displaced surgical-neck fractures that do not meet the exact displacement criteria of the Neer classification (1 cm or/and 45° angulation of displaced parts) where this reflects an individual surgeon’s equipoise (e.g. whether or not the surgical-neck fracture should be treated surgically)	
Exclusion criteria	Number of patients excluded^a^
1. Associated dislocation of the injured shoulder joint	101
2. Open fracture	2
3. Mentally incompetent patient: unable to understand trial procedure or instructions for rehabilitation; significant mental impairment that would preclude compliance with rehabilitation and treatment advice	116
4. Co-morbidities precluding surgery/anaesthesia	179
5. A clear indication for surgery such as severe soft-tissue compromise requiring surgery/emergency treatment (nerve injury/dysfunction)	87
6. Multiple injuries: same limb fractures; other upper limb fractures	72
7. Pathological fractures (other than osteoporotic)	5
8. Terminal illness	5
9. Participant not resident in trauma-centre catchment area	28

^a^More than one reason per patient possible

### Data collection and outcome measures

Lack of surgeon equipoise was ascertained based on information provided on the screening form under ‘other reason to exclude the patient’, following surgeon assessment against the formal trial inclusion/exclusion criteria (see Table 1). Where patients were excluded from the trial for such ‘other’ reasons alone, two independent raters (AR and HH) separately reviewed the reasons provided and came to a consensus on whether the exclusion was the result of a lack of surgeon equipoise. This was conducted as part of ongoing monitoring of recruitment; with concerns relating to excess lack of equipoise at individual hospitals being raised with the independent Trial Steering Committee (TSC) and subsequently with the hospital’s principal investigator (PI) if advised.

The wording of questions and response options relating to preferences for either trial treatment are detailed in Table 2. Surgeon preferences were collected for excluded patients, whereas surgeons were assumed to be in equipoise for the patients put forward for randomisation. For eligible but non-consenting patients, both surgeons and patients noted their treatment preferences. Randomised trial participants were asked for their treatment preferences at baseline and again at the end of the main trial follow-up at 2 years via the follow-up postal questionnaire.

**Table 2:** Patient and surgeon treatment preferences for different patient groups

		Surgeon preferences, *n* (%)					Patient preferences, *n* (%)					Agreed treatment, *n* (%)			
Patient group	Total *n*	Quest^1^	Surgery	Not surgery	Uncertain	Missing	Quest^1^	Surgery	Not surgery	No preference	Missing	Quest^1^	Surgery	Not surgery	Missing
Excluded (total)	687	*a*	232 (34%)	384 (56%)	–	71 (10%)	–	–	–	–	–	–	–	–	–
Excluded based on lack of equipoise	117	*a*	41 (35%)	58 (50%)	–	18 (15%)	*–*	–	–	–	–	–	–	–	–
Non-consenting	313	*b*	66 (21%)	105 (34%)	118 (38%)	24 (8%)	*c*	55 (18%)	226 (72%)	23 (7%)	9 (3%)	*d*	60 (19%)	242 (77%)	11 (4%)
Randomised	250	–	–	–	–	–	*e*	72 (29%)	60 (24%)	115 (46%)	3 (1%)	*f*	125 (50%)	125 (50%)	0 (0%)
Followed up at 2 years	218	–	–	–	–	–	*g*	86 (39%)	70 (32%)	54 (25%)	8 (4%)	–	–	–	–

^1^Key to wording of questions on Case Report Forms (CRFs):

*a* ‘What treatment would you advise for this patient?’ (Eligibility CRF)

*b* ‘Which treatment do you as the clinician advise the patient to have?’ (Consent Status CRF)

*c* ‘Does the patient express any treatment preference?’ (Consent Status CRF)

*d* ‘What is the agreed treatment for this patient?’ (Consent Status CRF)

*e* ‘In consenting for this trial, you have accepted that whether you get surgery or no surgery is left to chance. However, we would like to know if you had a preference before you agreed to this?’ (Baseline CRF)

*f* Agreed treatment as per randomisation

*g* ‘Based upon your experiences of the treatment that you received as part of this trial, if you injured your shoulder today to the same extent as you did 2 years ago, which treatment would you prefer?’ (2-year Follow-up Patient CRF)

Key patient characteristics collected for all patients at the eligibility assessment were: age, gender, affected shoulder (left or right), time since injury and tuberosity involvement (greater and/or lesser tuberosity). Age group (< 65 years versus ≥ 65 years) and fracture complexity (greater and/or lesser tuberosity versus neither tuberosity involved) were included as subgroup analyses in the main trial in line with surgeons’ expectations (younger patients and more complex fractures were expected to benefit more from surgery). We hypothesised that these characteristics may form the basis of preferences. These groupings were, therefore, included in all analyses; with age being analysed both as a continuous and dichotomised outcome.

Follow-up rates were calculated as the number of patient postal questionnaires returned at 3, 6, 12 and 24 months’ follow-up. Adherence to trial treatment was quantified as the proportion of patients who crossed over to the alternative treatment once they had been randomised, the uptake of physiotherapy and the patient-reported completion of home exercises. The latter two were recorded by the physiotherapists as part of the physiotherapy treatment logs.

The primary trial outcome was the Oxford Shoulder Score (OSS; scale 0–48, with higher scores indicating a better outcome) [16, 17]. It was collected via patient questionnaires at 6, 12 and 24 months. The trial was powered to detect a clinically important difference in the OSS of 5 points over 24 months. [9]

### Statistical analyses

All analyses were exploratory secondary analyses of the PROFHER trial data, and statistical tests were not formally powered. Statistical significance was accepted at the 0.05 level; however, consistent with criteria set for our subgroup analyses, the level for exploratory identification of possible confounding characteristics was set at *p* < 0.10. Stata Version 13.1 (StataCorp) was used for all analyses.

Treatment preferences expressed by surgeons and patients were tabulated for all patients screened for inclusion in the PROFHER trial, grouped by their eligibility and consent status. Baseline characteristics (age, gender, fracture complexity, affected shoulder and days since injury) were summarised for these populations. Comparisons were made between patient characteristics for four patient groups: those classified as being excluded due to lack of equipoise versus patients assessed as eligible; non-consenting versus randomised patients; surgeon preference for surgery versus not surgery among patients excluded due to lack of equipoise; patient preference for surgery versus not surgery among non-consenting patients. Comparisons were conducted using the independent *t* test (age), chi-squared tests (gender, age group, fracture type, affected shoulder) and Wilcoxon rank-sum test (days since injury). Treatment preferences of randomised patients at baseline and 24 months (where available) were cross-tabulated by allocated treatment arm and the pattern of change in treatment preferences described.

Differences in follow-up, crossovers, uptake of physiotherapy and uptake of home exercises were summarised for patients who did and did not receive their preferred treatment. To explore the impact of patient treatment preferences at baseline on OSS outcome differences, a preference by treatment interaction term was included in the primary analysis model, a mixed-effects model of OSS over 2 years’ follow-up. The model included as fixed effects: treatment group, time (6 months, 12 months or 24 months), treatment by time interaction, fracture type, age (< 65 years or ≥ 65 years), gender and health status (EuroQol five dimensions (EQ-5D) utility score) at baseline. Time points were nested within patients by means of an unstructured covariance structure. Any baseline characteristics that were found to be associated (*p* < 0.10) with either non-participation in the trial or preference for either treatment by surgeons or patients were included as an interaction term with the randomised trial treatment to the primary analysis. The interactions were plotted graphically, and the *p* values for the interactions reported. We note that analyses for interactions with age group and fracture type had been conducted as subgroup analyses of the main trial and are repeated here if relevant.

## Results

### Summary of recruitment and follow-up

Of 1250 patients screened, 563 (45%) were assessed as eligible based on the trial inclusion/exclusion criteria, of whom 250 (44%) were recruited and randomised. The rate of treatment crossovers was low: 16 (13%) of 125 patients allocated to surgery were treated non-surgically, and two (2%) of 125 patients allocated to non-surgical treatment received surgery. Completed patient questionnaires were obtained from 225 (90%) participants at 3 months, 232 (93%) at 6 months, 225 (90%) at 12 months and 215 (86%) at 24 months, with comparable return rates between treatment arms [10, 11].

### Surgeon preferences

**Fig. 1:**
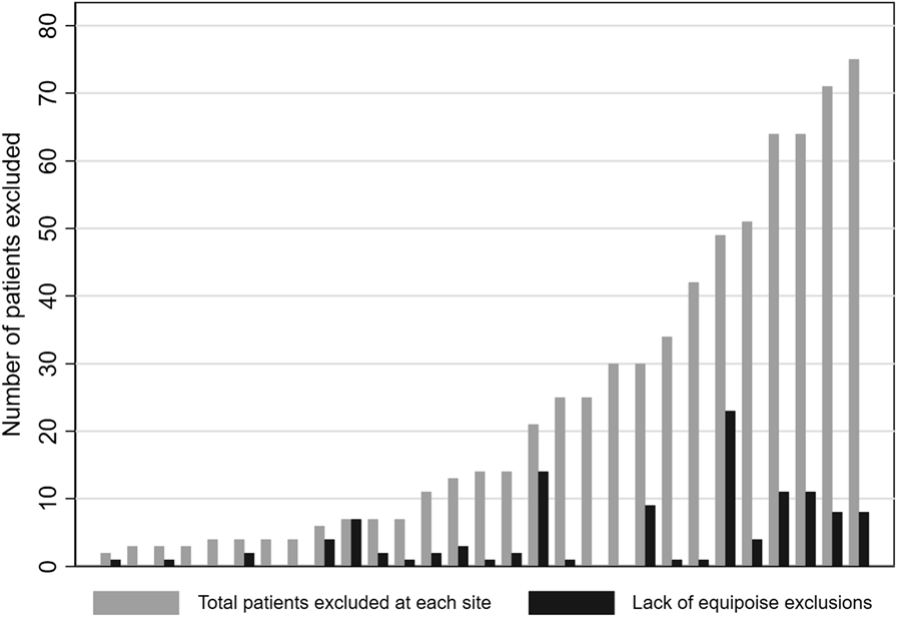
Lack of surgeon equipoise out of total excluded patients by centre, ordered by volume

The review of ‘other’ reasons for exclusion recorded by surgeons resulted in the identification of 117 patients (17% of the 687 excluded patients, also 17% of 680 otherwise eligible patients) who were excluded solely because of a lack of surgeon equipoise. Figure 1 illustrates their distribution by recruitment site, ordered by recruitment volume. Exclusions because of a lack of equipoise occurred to a varying extent in 22 centres and not at all in the remaining 11 centres. The PIs of the two centres with the highest number of inappropriately excluded patients (67% and 47% of excluded patients, respectively) were contacted with the agreement of the TSC and encouraged to review the trial protocol with participating surgeons. In a further notable centre, lack of equipoise was the reason for rejecting all of their seven excluded patients.

Lack of equipoise for one or more patients was recorded for 68 (38%) of 178 participating surgeons. Exclusion based on lack of equipoise was more prominent at some sites (12 of 17 surgeons in one centre) than others (1 in 16 in another). Twelve surgeons who showed lack of equipoise only screened and excluded one patient.

Compared with eligible patients, patients excluded based on lack of surgeon equipoise tended to be older (mean age: 69.9 years versus 67.4 years, *p* = 0.062; 72 vs 60% of age 65 years or over, *p* = 0.024) and were less likely to have a fracture involving one or both tuberosities (59 vs 75%, *p* < 0.001), see Table 3. Of 99 of these excluded patients for whom surgeons reported their advised treatment, 41 (41%) were advised surgery and 58 (59%) not surgery see (Table 2). Surgery was recommended more often for patients under the age of 65 years than ≥ 65 years (57 vs 36%, *p* = 0.059), and if the affected shoulder was the right one rather than the left one (50 vs 31%, *p* = 0.052), see Table 4. Fracture complexity did not appear to influence surgeon treatment recommendation (*p* = 0.794).

**Table 3:** Baseline characteristics for different PROximal Fracture of the Humerus Evaluation by Randomisation (PROFHER) patient populations

	Excluded patients			Eligible patients				
	A Excluded based on exclusion criteria or other valid reason	B Other sole reason for exclusion assessed to be lack of equipoise	C Total	D Non-consent	E Consented and randomised	F Total	*p* value for B vs F^1^	*p* value for D vs E^2^
Characteristic	(*N* = 570)	(*N* = 117)	(*N* = 687)	(*N* = 313)	(N = 250)	(*N* = 563)		
Gender								
Male, *n* (%)	160 (28%)	25 (21%)	185 (27%)	75 (24%)	58 (23%)	133 (24%)	0.586^3^	0.800^3^
Female, *n* (%)	404 (72%)	92 (79%)	496 (73%)	236 (76%)	192 (77%)	428 (76%)		
Missing, *n*	6	0	6	2	0	2		
Age (years)								
*N*	531	113	644	298	250	548		
Mean (SD)	70.6 (16.77)	69.9 (14.63)	70.5 (16.41)	68.5 (13.04)	66.0 (11.94)	67.4 (12.60)	0.062^4^	0.022^4^
Median	74.1	72.0	73.6	70.6	66.9	69.1		
< 65 years, *n* (%)	180 (34%)	32 (28%)	212 (33%)	109 (37%)	108 (43%)	217 (40%)	0.024^3^	0.114^3^
≥ 65 years, *n* (%)	351 (66%)	81 (72%)	432 (67%)	189 (63%)	142 (57%)	331 (60%)		
Missing, *n*	39	4	43	15	0	15		
Fracture type								
One or both tuberosities involved, *n* (%)	342 (60%)	69 (59%)	411 (60%)	229 (73%)	193 (77%)	422 (75%)	< 0.001^3^	0.272^3^
Neither tuberosity involved, *n* (%)	228 (40%)	48 (41%)	276 (40%)	84 (27%)	57 (23%)	141 (25%)		
Affected shoulder								
Left, *n* (%)	273 (51%)	62 (54%)	335 (51%)	160 (53%)	125 (50%)	285 (52%)	0.656^3^	0.486^3^
Right, *n* (%)	266 (49%)	53 (46%)	319 (49%)	142 (47%)	125 (50%)	267 (48%)		
Missing, *n*	31	2	33	11	0	11		
Days since injury								
*N*	570	117	687	313	250	563	0.853^5^	0.111^5^
Mean (SD)	4.9 (4.93)	5.6 (5.41)	5.0 (5.01)	5.2 (4.72)	5.7 (4.89)	5.4 (4.80)		
Median	3	3	3	4	4	4		

^1^Potential bias relating to surgeon preferences, ^2^Potential bias relating to patient preferences, ^3^Chi-squared test (*df* = 1), ^4^Independent *t* test, ^5^Wilcoxon rank-sum test. *SD* standard deviation

**Table 4:** Patient characteristics of patients excluded due to surgeon or patient preferences by preferred treatment

Characteristic	Surgeon preference (for patients excluded due to lack of equipoise, *n* = 117)			Patient preference (of non-consenting patients, *n* = 313)		
	Surgery	Not surgery	*p* value	Surgery	Not surgery	*p* value
Gender						
Male, *n* (%)	11 (48%)	12 (52%)	0.476^1^	11 (17%)	55 (83%)	0.476^1^
Female, *n* (%)	30 (39%)	46 (61%)		44 (21%)	169 (79%)	
Age (years)						
*N*	41	56		53	214	
Mean (SD)	68.0 (2.06)	72.4 (1.76)	0.105^2^	65.1 (15.59)	69.2 (12.21)	0.039^2^
Median	68.8	75.0		69.3	70.7	
< 65 years, *n* (%)	16 (57%)	12 (43%)	0.059^1^	23 (23%)	77 (77%)	0.318^1^
≥ 65 years, *n* (%)	25 (36%)	44 (64%)		30 (18%)	137 (82%)	
Fracture type						
One or both tuberosities involved, *n* (%)	23 (43%)	31 (57%)	0.794^1^	44 (22%)	160 (78%)	0.170^1^
Neither tuberosity involved, *n*	18 (40%)	27 (60%)		11 (14%)	66 (86%)	
Affected shoulder						
Left, *n* (%)	15 (31%)	34 (69%)	0.052^1^	28 (20%)	109 (80%)	0.953^1^
Right, *n* (%)	24 (50%)	24 (50%)		27 (20%)	107 (80%)	
Days since injury						
*N*	41	58		55	226	
Mean (SD)	6.5 (5.83)	4.7 (4.94)	0.253^3^	5.5 (5.26)	5.2 (4.68)	0.912^3^
Median	4	2		4	3	

^1^Chi-squared test (*df* = 1), ^2^Independent *t* test, ^3^Wilcoxon rank-sum test. *SD* standard deviation

### Patient preferences

Of 563 eligible patients, 313 (56%) did not consent to take part in the trial. Compared with consenting patients, non-consenters tended to be slightly older (mean age: 68.5 years versus 66.0 years, *p* = 0.022; however, our a priori age threshold of 65 years appeared less relevant to patients: *p* = 0.114), see Table 3. Fracture severity did not affect patient preferences (*p* = 0.272). Of 304 non-consenting patients who indicated a treatment preference, the majority (*n* = 226, 74%) preferred not surgery, whereas 55 (18%) preferred surgery and 23 (8%) had no preference, see Table 2.

Younger patients were more likely to favour surgery than not surgery (mean of 65.1 years versus 69.2 years, *p* = 0.039); however, this was not reflected by patients being below or above 65 years of age (*p* = 0.318), see Table 4. Fracture type in terms of tuberosity involvement did not appear to affect treatment choice to a significant extent (*p* = 0.170), although the trend was for surgery to be preferable for more complex fractures than less complex ones (44/204; 22 vs 11/77; 14%).

At 24 months’ follow-up, patients who received their preferred treatment largely retained their baseline preference, which was stronger for non-surgery (20/28; 71%) than surgery (12/31; 61%), see Table 5. Patients who did not receive their preferred treatment were more likely to change their preference (38/63, 60%) compared with those who did (18/69, 26%). These patients changed their preference to their allocated treatment to a similar extent (38% in the surgery arm and 39% in the non-surgery arm). However, patients with non-preferred allocation were more likely to retain their original preference if this was not surgery (8/32; 25% vs 5/31; 16% for surgery). Patients with non-preferred allocation who initially preferred surgery were more likely to change to no preference (9/31; 29% vs 5/32; 16% for not surgery). Of those patients who had no preference at baseline, more surgery patients preferred their allocated treatment at 24 months (27/52; 52%) compared with not-surgery patients (22/63; 35%).

**Table 5:** Treatment preferences of randomised participants at baseline and 24-month follow-up by treatment allocation

		Patient preference at baseline^a^ Randomised allocation (*n*)					
		Surgery		Not surgery		No preference	
Patient preference at 24 months		Surgery	Not surgery	Surgery	Not surgery	Surgery	Not surgery
		(*n* = 41)	(*n* = 31)	(*n* = 32)	(*n* = 28)	(*n* = 52)	(*n* = 63)
Surgery, *n* (%)		25 (61%)	5 (16%)	12 (38%)	3 (11%)	27 (52%)	12 (19%)
Not surgery, *n* (%)		4 (10%)	12 (39%)	8 (25%)	20 (71%)	3 (6%)	22 (35%)
No preference, *n* (%)		7 (17%)	9 (29%)	5 (16%)	4 (14%)	12 (23%)	17 (27%)
Missing, *n* (%)		5 (12%)	5 (16%)	7 (22%)	1 (4%)	10 (19%)	12 (19%)

^a^Patients with missing baseline preferences are excluded from this table (*n* = 3)

### Impact of preferences on PROFHER follow-up and treatment adherence

Overall return rates of patient-completed postal questionnaires were high; however, patients randomised to their preferred treatment were more likely to return questionnaires (97%, 96%, 94% and 94% at 3 months, 6 months, 12 months and 24 months, respectively) compared with patients randomised to their non-preferred treatment (87%, 94%, 90% and 84%) or patients who had no treatment preferences (87%, 90%, 88% and 84%). Of the 18 treatment crossovers in the trial, 9 (50%, 8 surgery, 1 non-surgery) could be attributed to patient preference (reported as change of mind, 8 of these had been allocated to their non-preferred treatment) and 3 (17%, 2 surgery, 1 non-surgery) could be attributed to surgeon preferences (change of mind or preference by a different surgeon). The remaining six (33%) crossovers were patients given non-surgical treatment after being identified as unfit for surgery. Patients who were allocated to their preferred treatment took up the physiotherapy element of treatment to a similar extent (96% started treatment, median of eight sessions) compared with patients who were not allocated to their preferred treatment (95% started treatment, median of eight sessions) and patients who had no preference (93% started treatment, median of 7.5 sessions). Home exercises were self-reported as being completed by 90%, 84% and 82% of patients who were allocated to their preferred treatment (total *n* = 69), non-preferred treatment (*n* = 63) and with no preference, respectively (*n* = 115).

### Impact of preferences on PROFHER primary outcome

**Fig. 2:**
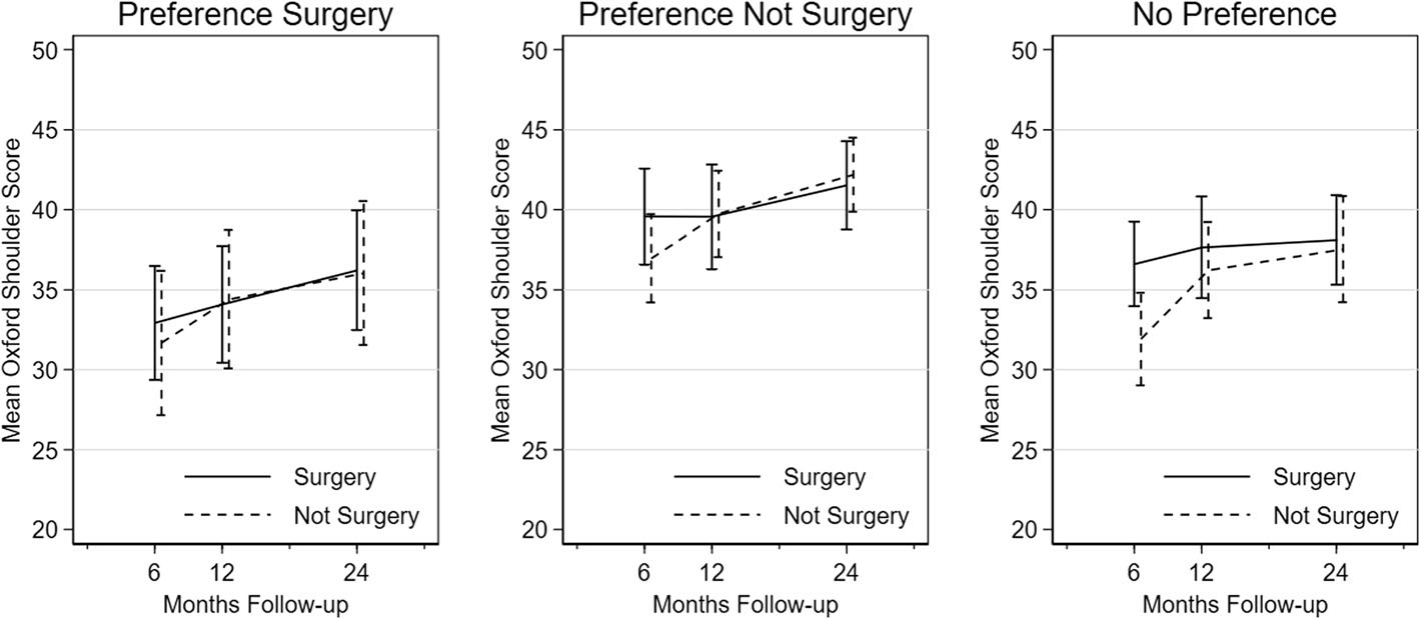
Comparison of Oxford Shoulder Score by treatment arm, grouped by patient baseline preferences

**Fig. 3:**
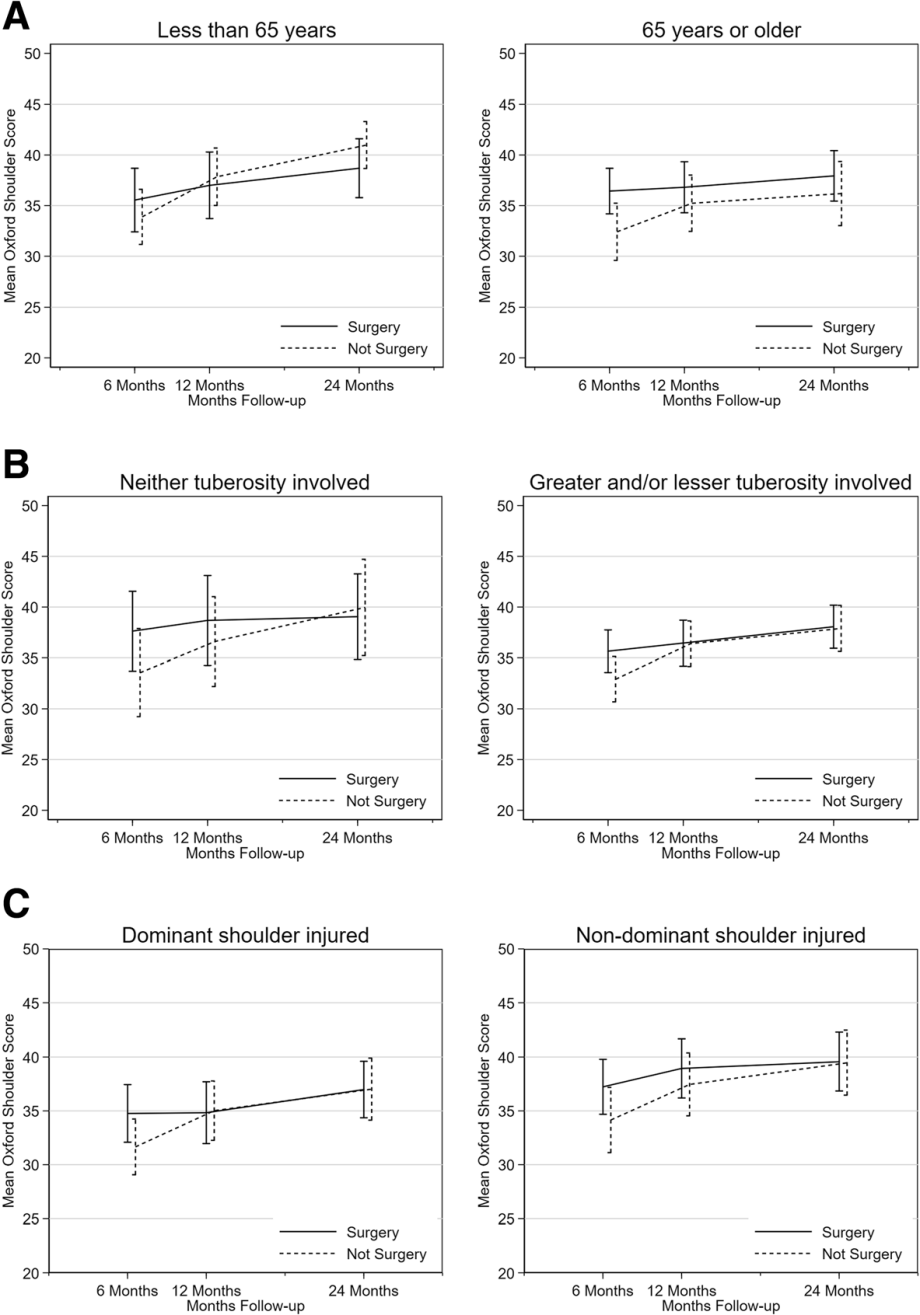
Comparison of Oxford Shoulder Score by treatment arm, by subgroups of age (**a**), fracture type (**b**) and shoulder dominance (**c**)

OSS outcomes by patient preference at baseline are illustrated in Fig. 2. Patients who preferred not surgery generally had higher OSS scores, indicating greater functioning, than patients who preferred surgery. However, there were no treatment-group differences based on baseline preference (*p* value of interaction = 0.751). In terms of characteristics associated with treatment preferences, age was shown to impact on the selection of trial patients by surgeons into the trial (older patients were more likely to be excluded) and to influence treatment choice by surgeons and patients (surgery being favoured for and by younger patients). The PROFHER subgroup analysis by age group demonstrated, however, that the pattern of OSS scores following surgical or non-surgical treatment did not differ for patients above or below 65 years of age (*p* value of interaction = 0.264), see Fig. 3. Furthermore, fracture complexity played a role in decision making for surgeons who were not in equipoise (fractures involving neither tuberosities being more likely to be excluded). Again, the subgroup analysis by fracture type demonstrated that treatment-group differences in OSS scores did not differ for tuberosity involvement (*p* value of interaction = 0.954). In addition to subgroup analyses that had already been conducted, OSS scores were compared between patients who injured their dominant and non-dominant shoulder, following the observation that surgeons not in equipoise were more likely to recommend surgery when the right shoulder was affected. While shoulder dominance was not collected for excluded patients, the side of injury and dominance were synonymous for 89% of randomised participants. Results show that patients who injured their dominant shoulder had on average worse outcomes; however, the differences between surgical and non-surgical treatment were comparable for dominant and non-dominant shoulders (*p* value of interaction = 0.850), see Fig. 3.

## Discussion

### Summary of results

In our exploration of the effects of surgeon and patient treatment preferences on bias and applicability in the PROFHER trial, we have addressed all of our study objectives. In terms of surgeon treatment preferences and with reference to our objectives, we found that (a) 17% of otherwise eligible patients were excluded because of lack of surgeon equipoise; and these excluded patients were on average 2.5 years older and had less complex fractures (no tuberosity involvement) than eligible patients and (b) surgeons tended to preferentially recommend surgery for younger patients and patients who had injured their right shoulder, but that fracture type did not seem to determine treatment recommendation.

In terms of patient preferences, we found that (c) over half of otherwise eligible patients (56%) did not consent to take part in the trial, and non-consenters were on average 2.5 years older and preferred not surgery (74%); (d) preference for surgery or not surgery by non-consenters was not associated with age or any other baseline characteristics and (e) at 24 months’ follow-up, similar proportions of patients switched their treatment preference to their allocated treatment in each trial arm; however, for patients who were not allocated to their preferred treatment, individuals with a baseline preference for surgery were more likely to change to no preference than patients who preferred not surgery.

Finally, in our examination of the impact of preferences we found that (f) for patients who were allocated their preferred treatment slightly better follow-up rates, fewer patients who changed their mind regarding their willingness to receive their randomised treatment and slightly greater engagement with therapeutic elements such as completing home exercises; (g) in terms of the PROFHER primary outcome (OSS), that there were no treatment-group differences based on patient preferences at baseline and that (h) subgroups based on factors linked to patient selection or treatment preferences (age, tuberosity involvement and shoulder dominance) did not show differential response to surgical and non-surgical treatment.

The research arena before PROFHER, as for several other recently undertaken NIHR-funded, substantial multi-centre RCTs in orthopaedic trauma surgery research such as DRAFFT [18], was of poor quality, featuring small, often under-recruited RCTs and aborted trials [9]. Achievement of our recruitment target alone is an indication of success in meeting the challenges of surgeon and patient treatment preferences, albeit over a more extended time period than planned (31 versus 18 months). Other substantive indicators are low crossover rates, high retention rates and comparable standards in care including equivalent participation in home exercises in both treatment groups. The findings of our exploratory analyses of the effects of treatment preferences should be seen in this context.

### Surgeon treatment preferences

Although lack of surgeon equipoise is often considered an influential contributor to poor trial recruitment, we have found very few estimates in the literature of the extent of loss of otherwise eligible patients attributed specifically to this. We note, however, a similar lack of surgeon equipoise accounted for the loss of 17% of all potentially eligible patients in the Australasian Laparoscopic Colon Cancer Study [19]. Our finding that excluded eligible patients tended to be older is consistent with surgery being recommended more often for younger patients; this reflects current practice and that patient age has been demonstrated to affect surgeons’ treatment decisions for these fractures [20]. Our examination of the centre distribution of excluded patients because of lack of equipoise showed an uneven distribution, with nearly a third of exclusions from just two centres and none from 11 centres. The potential of centre-specific effects is countered by the confirmation of no statistically significant treatment difference following adjustment for clustering by centre in a sensitivity analysis of the primary outcome [11]. The finding that 68 of 178 surgeons in PROFHER excluded one or more otherwise eligible patients due to lack of equipoise is concerning in terms of the perceived applicability of the trial results. Ziebland et al. in the Spine Stabilisation Trial reported the underlining lack of understanding of the study inclusion criteria and design by such surgeons could lead to a reluctance to apply the results to their own practice [21]. Nonetheless, the PROFHER trial population has been independently assessed as representative of current UK practice [12], and the results of a survey of 265 orthopaedic shoulder surgeons in 2016 demonstrated notable changes in UK practice in line with, and because of, PROFHER findings [22].

### Patient treatment preferences

Nearly three quarters (72%) of non-consenting patients preferred not surgery, with only 7% having no preference. In contrast, slightly more consenting patients preferred surgery (29%) than non-surgery (24%), and almost half (46%) had no treatment preference at baseline. These findings are consistent with our expectations for this patient group, both in terms of a general preference for not surgery and also a greater proportion of patients with no preference among those willing to be randomised. The involvement of impartial research nurses or physiotherapists, rather than the treating surgeons, at the consent phase may well have contributed to the lack of treatment preference, as did the training providing by the trial team and development of a patient information leaflet with patient representative input. We note that retention and adherence to the trial treatments were already high in PROFHER and balanced between treatment arms. Although the differences observed here were in line with previous findings, which tended to show that those without a treatment preference had the lowest response rates during follow-up [5], we note that they were of small magnitude in terms of patient numbers with little scope to impact on the study results. Crucially, patient treatment preferences did not significantly affect patients’ treatment outcome with regard to the main OSS findings.

### Limitations

As noted in our methods, all statistical tests were not formally powered; however, there were no emerging trends for preference-based treatment differences. While our study adjusted the trial analysis for differences in the composition of included and excluded patient populations based on preferences in order to confirm the external validity of the PROFHER findings, we acknowledge that populations may have differed in other unknown aspects that we could not control for. However, we note that we included major a priori determinants of patient selection such as age and fracture complexity.

Potential anomalies can arise from the use of artificial thresholds for continuous outcomes. Our selection of the 65-year age boundary is one that reflects a commonly perceived threshold for older people by clinicians that was likely to form part of their decision making. It is not surprising that patients were less influenced by this cut-off, as this was unlikely to be part of their considerations. Also notable is that readiness or acceptability for surgery is likely to be condition dependent; the design of a trial investigating anterior cruciate ligament reconstruction surgery reflected the strong preferences in younger patients for surgery [23].

At the time of finalising our trial design, we accepted our funder’s instruction and guidance not to perform a comprehensive cohort design as it was likely to distract from RCT recruitment nor to undertake a qualitative study to explore patients’ reasons for non-participation in the trial. (We also accepted Ethics Committee guidance not to ask non-consenting patients for their reasons for non-participation.). These methods have provided useful insights on treatment preferences in other areas [19, 24, 25]. However, as shown by our paper, our systematic approach is feasible, has provided valuable insights and, we suggest, is one suitable for wider adoption.

## Conclusions

Although PROFHER recruited to target, the need for a recruitment extension was, in part, the result of the preferences held by surgeons (lack of equipoise) and patients (lack of consent). A systematic multicomponent exploration of the extent and impact of surgeon and patient preferences revealed a consistent picture, primarily where preferences for not surgery by surgeons and patients alike were associated with older patient age, which led to slightly younger patients being recruited into the trial. Secondary analyses showed that neither surgeon nor patient treatment preferences importantly affected internal validity (differences in dropout and adherence were of small magnitude in light of the overwhelmingly high follow-up and engagement with the interventions in PROFHER) nor external validity (no meaningful treatment differences for predictors of patient selection, including age, and treatment preferences). Independent corroboration of the external validity of the trial findings was via the inclusion of these in national guidance (NICE) as well as separate evidence of acceptance of trial findings by specialist UK surgeons. Ongoing monitoring of the lack of equipoise at sites helped to manage surgeon bias in the trial, and the collection of data presented here proved invaluable in evaluating the validity of the trial results.

## Data Availability

The data analysed for the present study are available via the corresponding author on reasonable request to York Trials Unit.

## Abbreviations

CRF: Case Report Form
NICE: National Institute for Health and Care Excellence
OSS: Oxford Shoulder Score
PI: Principal investigator
PROFHER: PROximal Fracture of the Humerus Evaluation by Randomisation
RCT: Randomised controlled trial
TSC: Trial Steering Committee
UK: United Kingdom
